# HumanaFly: high-throughput transgenesis and expression of breast cancer transcripts in *Drosophila* eye discovers the RPS12-Wingless signaling axis

**DOI:** 10.1038/s41598-020-77942-x

**Published:** 2020-12-03

**Authors:** Vladimir L. Katanaev, Mikhail Kryuchkov, Volodymyr Averkov, Mikhail Savitsky, Kseniya Nikolaeva, Nadezhda Klimova, Sergei Khaustov, Gonzalo P. Solis

**Affiliations:** 1 Translational Research Center in Oncohaematology, Department of Cell Physiology and Metabolism, Faculty of Medicine, University of Geneva, Geneva, Switzerland; 2 Developmental Genetics Group, Institute of Protein Research, Russian Academy of Sciences, Pushchino, Moscow Region Russia; 3 School of Biomedicine, Far Eastern Federal University, Vladivostok, Russia

**Keywords:** Breast cancer, Development, Cell signalling

## Abstract

*Drosophila melanogaster* has been a model for multiple human disease conditions, including cancer. Among *Drosophila* tissues, the eye development is particularly sensitive to perturbations of the embryonic signaling pathways, whose improper activation in humans underlies various forms of cancer. We have launched the HumanaFly project, whereas human genes expressed in breast cancer patients are screened for their ability to aberrate development of the *Drosophila* eye, hoping to thus identify novel oncogenes. Here we report identification of a breast cancer transgene, which upon expression in *Drosophila* produces eye malformation similar to the famous *Glazed* phenotype discovered by Thomas Morgan and decades later dissected to originate from mis-expression of Wingless (Wg). Wg is the ortholog of human Wnt proteins serving as ligands to initiate the developmental/oncogenic Wnt signaling pathway. Through genetic experiments we identified that this transgene interacted with the Wg production machinery, rather than with Wg signal transduction. In *Drosophila* imaginal discs, we directly show that the transgene promoted long-range diffusion of Wg, affecting expression of the Wg target genes. The transgene emerged to encode RPS12—a protein of the small ribosomal subunit overexpressed in several cancer types and known to also possess extra-ribosomal functions. Our work identifies RPS12 as an unexpected regulator of secretion and activity of Wnts. As Wnt signaling is particularly important in the context of breast cancer initiation and progression, RPS12 might be implicated in tumorigenesis in this and other Wnt-dependent cancers. Continuation of our HumanaFly project may bring further discoveries on oncogenic mechanisms.

## Introduction

Despite the ‘war on cancer’ proclaimed back in 1970s, tumors continue to occupy the second place in the disease-related death toll in developed countries. Cancer originates from—and in its progression further accumulates—multifaceted dysregulations of cellular and tissue activities. Among such dysregulations, signaling pathways that control cell proliferation and differentiation during embryonic development but are largely silent in the adult become aberrantly reactivated to drive oncogenic transformation and progression. Although several key players within these oncogenic pathways have been identified as targets for the ongoing drug discovery, understanding of the mechanistic details of how such pathways are abused in cancer is still incomplete.

Driven by the inherent kinship of the embryonic and oncogenic signaling pathways, as well as by the fact that around 75% of pathology-related human genes have orthologues in *Drosophila*^1^ making this fruit fly such a powerful model for various human maladies, we have launched the HumanaFly project. In this project, mRNAs isolated from human breast cancer biopsy are cloned in a vector for *Drosophila* germ-line integration and expression in the developing eye of the insect. This organ requires intricate interplay of all the major embryonic/oncogenic pathways for its proper development, and the genetics of *Drosophila* eye malformation has been the source of elucidation of the architecture of such pathways, conserved from insects to humans^2^. Expression of human oncogenes (Ras-like GTPase Ral, Cyclin D1/Cdk4 complex, anaplastic lymphoma receptor tyrosine kinase ALK, chromatin regulator DEK, negative regulator of apoptosis Bcl-2, acute myeloid leukemia’s BCR-ABL, and more) in *Drosophila* eyes has previously been described to induce strong malformations of the organ^3–8^, providing positive examples ahead of our HumanaFly project.

The Wnt signaling is one of the major embryonic/oncogenic pathways. In embryogenesis, it regulates numerous developmental patterning and differentiation programs, controlling in *Drosophila* the eye and wing development among others. In the developing eye, Wingless (Wg, *Drosophila* Wnt1) is produced by the periphery of the eye imaginal disc, mediating formation of the head capsule and diffusing into the disc proper to create a gradient responsible for the patterning and polarity of the differentiated eye cells^9^. In the wing, Wg is secreted by a narrow stripe of cells at the dorso-ventral boundary of the wing imaginal disc; its diffusion through the wing disc also creates a concentration gradient responsible for the tissue patterning^10^. In adult humans, the Wnt pathway is mostly silent and its reactivation in various tissues such as the female breast causes oncogenic transformation; Wnt signaling is particularly important for the deadliest tumor of this tissue, the triple-negative breast cancer (TNBC) taking annually some quarter of a million lives on the global scale and currently lacking any targeted therapies^11,12^. Wg and other Wnt ligands are lipoglycoproteins secreted from the producing cells by a sophisticated machinery ensuring proper diffusion of these morphogens though the receiving tissues^13^. Two (or more) distinct pools of Wnts are believed to be secreted, differing by the vehicle in which these hydrophobic proteins are packed. Monomeric (or small oligomeric) Wnts are destined for the short-range diffusion, creating a high concentration close to the source of production and turning on the short-range target genes. In contrast, packaging of Wnts into lipoprotein particles or liposomes hindering the hydrophobic moieties of the morphogens creates the long-range diffusion form. The reggie-1/flotillin-2 protein has been identified as a ‘pointsman’ in the Wnt-producing cells, directing Wnt secretion into one or the other route for the short- *vs.* long-range diffusion^14^.

About 80 ribosomal proteins, in addition to the ribosomal RNAs, constitute the large and the small subunits of the ribosome—the protein building factory of our cells^15^. Cancer cells typically display increased metabolic and cell-division activities, which are procured by increased ribosomal content^16^. In addition to that, several ribosomal proteins have been found to possess extra-ribosomal activities, relevant both for the healthy and pathological cellular states^17,18^. In the run of the HumanaFly project, we identify the TNBC transgene encoding the ribosomal protein RPS12 to cause *Drosophila* eye malformations similar to those observed upon Wg overexpression. In a set of experiments in the developing eye and wing of the fruit fly, we show that RPS12 promotes long-range diffusion of Wg, emerging as an unexpected regulator of secretion and activity of Wnt proteins. Given the primary importance of Wnt signaling in TNBC, our studies may have uncovered a potential novel target for anticancer drug discovery.

## Materials and methods

Total RNA (6 μg) was isolated from the surgical resect of a TNBC cancer patient (grade 3 infiltrating ductal carcinoma, 61 years-old post-menopausal Caucasian female) by Asterand Bioscience, United Kingdom (case ID 17995), and poly(A)-containing mRNAs were reverse-transcribed and cloned into the pUASTattB plasmid by Dualsystems, Switzerland.

The DNA library was germ-line transformed into the *yw, ZH-attP-22A (φχ22A) Drosophila* host line^19^. P0 flies were crossed to the *yw; CyO/Sp; GMR-Gal4/GMR-Gal4* line at 23.5 °C. In the P1, the red eye coloration reflected successful germ-line transformation, while eye roughness (or other visible eye malformations) were indicative of the eye development aberration caused by the cancer transgene expression under the *GMR-Gal4* control, driving expression starting from the moment of eye cell differentiation^2^. These lines were further balanced and maintained as *yw; UAS-transgene/CyO; GMR-Gal4/TM6B*.

Other *Drosophila* lines used in this study were: *dRPS12*^*s2783*^*/* + (Bloomington), *UAS-RNAi-dRPS12* (VDRC ID109381) and *UAS-RNAi-Wg* (VDRC ID13351, both from Vienna *Drosophila* RNAi Center), *UAS-AxinΔRGS*^20,21^, *engrailed-Gal4; UAS-GFP*^22^, *hedgehog-Gal4*^23^, and *wg-Gal4* (ND382)^24,25^.

Post single-fly phenol–chloroform DNA extraction, PCR-amplification of the transgene was achieved with oligonucleotides specific to the pUASTattB vector, pUAST320for (GTAACCAGCAACCAAGTA) and pUAST505rev (GTCCAATTATGTCACACC), and sequenced.

Adult *Drosophila* eyes were photographed through a binocular equipped with a digital camera. Relative eye areas were calculated with Photoshop. Adult eye cross-sections were performed as described^26^. Atomic-force microscopy examination of corneal nanocoatings was performed as described^27^. Late 3rd-instar larval imaginal discs (eye-antennal and wing) were dissected and immuno-stained for confocal microscopy as described^14^. For the extracellular Wg staining, the following protocol was employed: discs were incubated 30 min in PBS with anti-Wg antibodies on ice (without permeabilization), washed with PBS and fixed with 4% paraformaldehyde in PBS. After fixation the discs were washed with PBS, permeabilized with 0.5% NP40, then washed again and immunostained in 0.2% Tween 20 in PBS with anti-hRPS12. After washing with 0.2% Tween 20 in PBS, the discs were incubated with secondary antibodies against both primary antibodies. The following antibodies were used: rabbit anti-hRPS12 at 1:100 from Proteintech (Cat: 16490–1-AP), mouse anti-Wg (clone 4D4) at 1:50 (1:10 for extracellular staining) from Developmental Studies Hybridoma Bank, guinea pig anti-Sens at 1:1000 dilution^28^. FITC-, Cy3- and Cy5-labeled (Jackson ImmunoResearch) secondary antibodies were used.

The phenotypes shown on Figs. 2, 4, and 5 are 100% penetrant. Unpaired t-test was used to probe statistical significance in Fig. 3.

## Results

### Early-phase of the HumanaFly project (Fig. 1)

**Figure 1:**
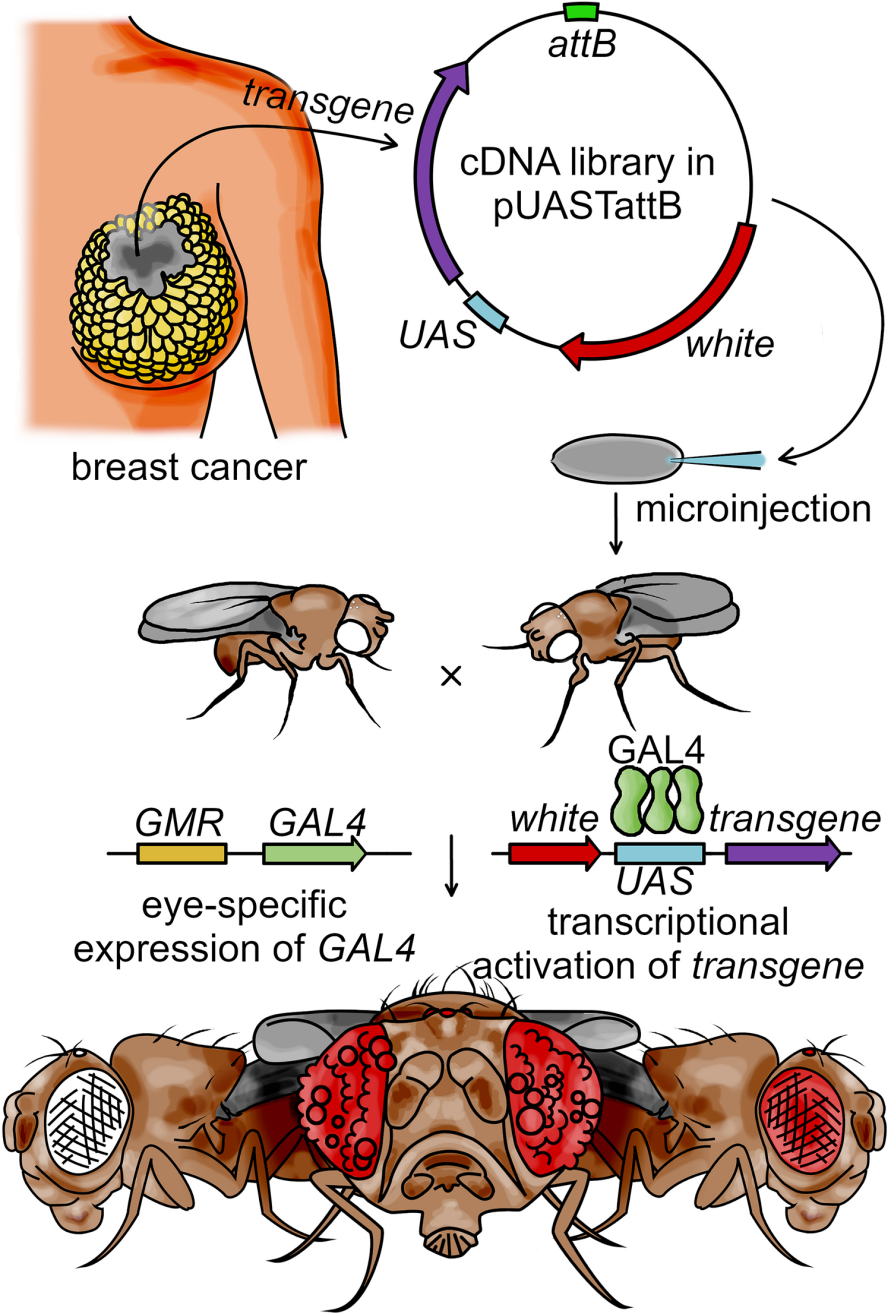
Scheme of the HumanaFly project. mRNAs isolated from a patient-derived triple-negative breast cancer are reverse-transcribed and re-cloned into a plasmid for germ-line *Drosophila* transformation and subsequent *Gal4-UAS*-mediated expression in *Drosophila* eyes. Transformants are identified by red eye coloration, and cancer transcripts whose expression in the eye drives developmental malformation are selected for further investigation.

mRNA isolated from infiltrating ductal carcinoma of a triple-negative breast cancer (TNBC) patient was reverse-transcribed and re-cloned into the pUASTattB vector for germ-line transformation into the *ZH-attP-22A (φχ22A) Drosophila* line. This way, all the transgenes landed into the same 22A site of the chromosome arm 2R, excluding any locus-dependent variability in the resultant transgene expression levels. While the recipient line is *white*^*-*^ with white-colored eyes (Fig. 1, Supplementary Fig. 1A), the pUASTattB vector carries the *white*^+^ transgene as a marker, marking the successful transformants with red-colored eyes (Fig. 1, Supplementary Fig. 1B).

**Figure 2:**
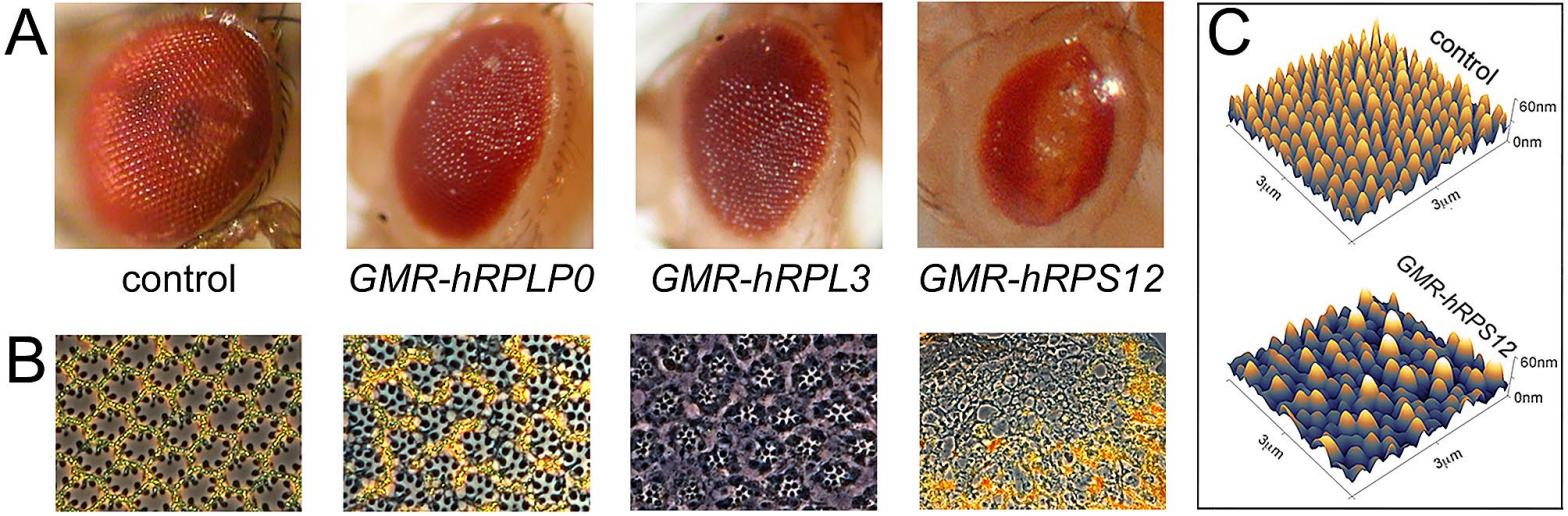
Human ribosomal proteins induce malformations in *Drosophila* eyes. (**A**) Expression of human RPLP0 and RPL3 under the *GMR-Gal4* control produces the rough eye phenotype, while hRPS12 induces a glossy eye of a reduced size reminiscent of the *Glazed* mutation. (**B**) Cross-sections of these eyes shows partial pigment cell and photoreceptor degeneration in the rough eyes, with massive cell degeneration and ommatidial fusion in *GMR-hRPS12* eyes. (C) Atomic force microscopy examination of corneal surfaces in wild-type and *GMR-hRPS12* eyes. Scanning area is 3 × 3 μm.

The post-injection P0 flies were crossed with the line *yw; CyO/Sp; GMR-Gal4/GMR-Gal4*. This permitted not only identifying the individual transformant lines in the P1, but also selecting for the lines whose *GMR*-driven transgene expression produced eye malformations. In this manner, the initial round of the HumanaFly project produced ca. 2′000 transformants, of which 6 depicted various degrees of eye roughness or malformation. PCR-amplification and sequencing of the transgenes from the 6 lines identified them to encode ANKRD17 and OXA1L (Supplementary Fig. 1C,D), as well as human ribosomal proteins RPLP0, RPL3 (present in 2 lines), and RPS12 (Fig. 2A). Randomly picked non-rough eye transformants were identified to encode DENND1A and another ribosomal protein RPS23 (not shown). The frequent encounter of the human ribosomal transgenes among the transformants is not surprising given the abundance of their mRNAs. However, non-ribosomal transgenes were also identified. This initial phase of realization of the HumanaFly project suggested that the approach we adopted was functional to identify human cancer mRNAs whose mis-expression in the developing eye of *Drosophila* led to malformation of this organ. While the project is still ongoing, we here concentrated on the effects of the human ribosomal proteins in *Drosophila* eye, given the unusual phenotypes induced by RPS12 (see below).

### Human ribosomal genes produce different eye malformations in *Drosophila*

Of the three human ribosomal proteins, whose production in the developing *Drosophila* eye induced malformations in the organ, two (RPLP0 and RPL3) produced eye roughness, while one—RPS12—induced the phenotype with reduced eye size and the overall glossy appearance of the eye surface (Fig. 2A). Such a phenotype is reminiscent of the famous *Glazed* mutant discovered in 1936 by the father of *Drosophila* genetics, Thomas Morgan, resulting from photoreceptor degeneration, ommatidial fusion and degeneration of the corneal nanostructures decorating the lens surface and normally serving to minimize reflection of the incoming light^27,29^. We thus performed the ultrastructural analysis of retinal cross-sections of the mutant eyes. This analysis shows that while expression of human RPLP0 (*GMR-hRPLP0*) leads to partial degeneration of pigment cells and of human RPL3 (*GMR-hRPL3*) —to partial photoreceptor degeneration, expression of human RPS12 (*GMR-hRPS12*) drives strong ommatidial degeneration and fusion (Fig. 2B), very much like in the *Glazed* mutants^29^. We further showed that the *GMR-hRPS12* eye cornea are strongly devoid of the regular nipple-type anti-reflective nanocoatings (Fig. 2C), again similar to the *Glazed* eyes^27^; no other transformant produced the deregulation of the corneal nipple arrays (data not shown).

**Figure 3:**
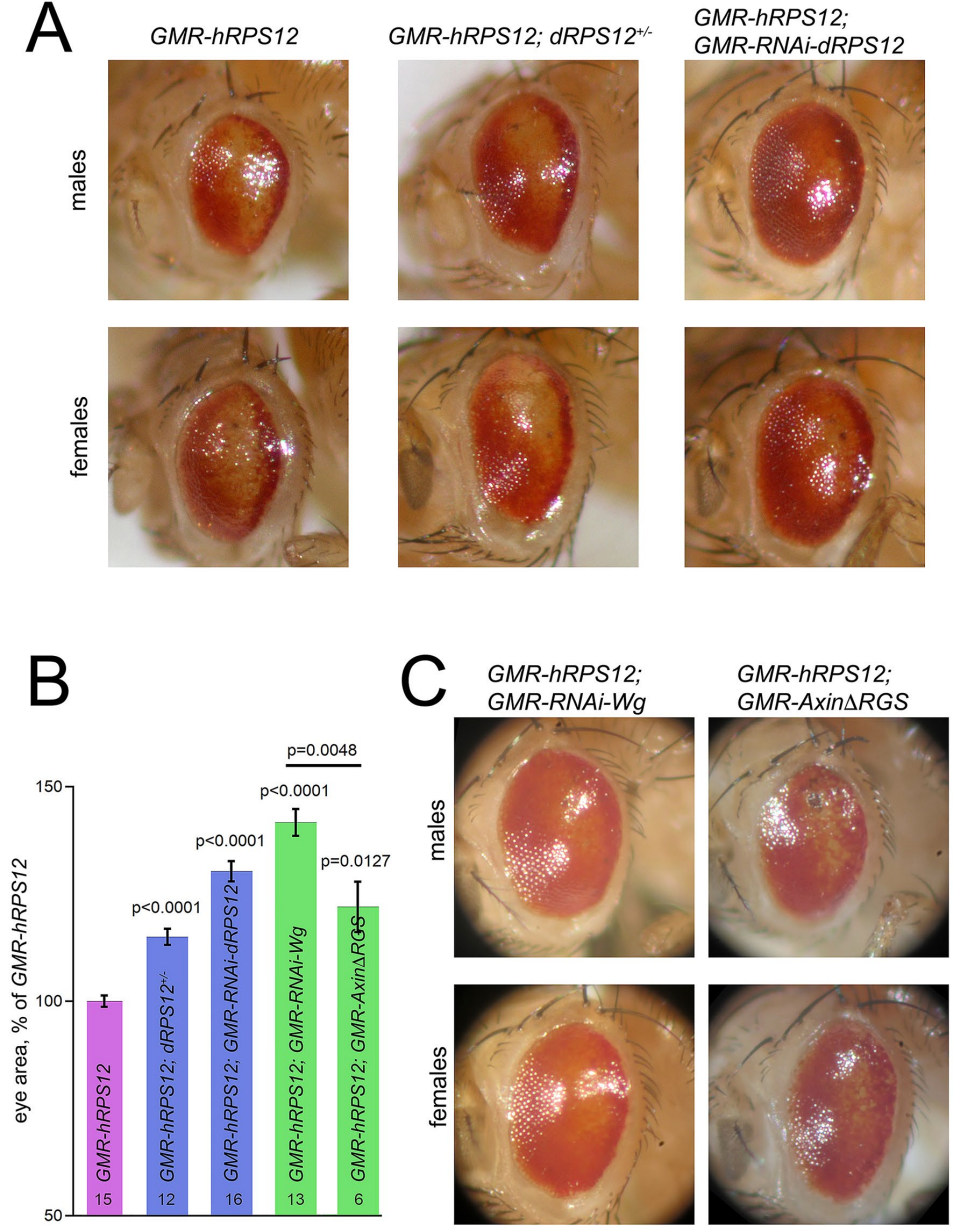
The *GMR-hRPS12* phenotype is partially rescued by reduced levels of endogenous *Drosophila* RPS12 and Wg. (**A**) The *Glazed*-like phenotype of *GMR-hRPS12* is partially rescued by removal of one gene copy of *dRPS12* or by co-expression of an RNAi against *dRPS12*. Representative eyes of male and female flies (naturally different in size) are shown. (**B**) Quantification of the genetic interactions of *GMR-hRPS12*. Area size of eyes of different genotypes was quantified, separately for male and female flies. The eye size of *GMR-hRPS12* flies was taken as 100% (pink bar). The effect of genetic modifications introduced on top of the *GMR-hRPS12* genotype was then quantified as the increase in the eye size over *GMR-hRPS12* flies. Decrease in endogenous dRPS12 levels (blue bars) and in Wg signaling (green bars) both partially rescue the *GMR-hRPS12* phenotype. Number of eyes analyzed is given at the bottom of each bar. Unpaired t-test was used to probe statistical significance of the eye area difference from the *GMR-hRPS12* phenotype (as well as between the two genotypes marked in green); the resulting p-value is given above each bar. (**C**) The *GMR-hRPS12* phenotype is partially rescued by co-expression of an RNAi against *Wg*, but much less—by co-expression of AxinΔRGS inhibiting Wg signaling. Quantification is shown in (**B**).

We found that the *GMR-hRPS12* phenotypes are partially suppressed by reducing the dosage of *Drosophila* RPS12 (dRPS12). Both in male and female flies, expression of hRPS12 on the background heterozygous for the loss-of-function mutation in the endogenous *dRPS12* gene produced less severe phenotypes (Fig. 3A,B). Similarly, co-expression of an RNAi construct specific to the endogenous *Drosophila* RPS12 alleviated the eye size reduction and ommatidial fusion induced by hRPS12 (Fig. 3A,B). Thus, human and *Drosophila* RPS12 proteins can at least partially replace each other.

### Genetic interactions of human RPS12 and Wg

Only 60 years after the Morgan’s description of the *Glazed* mutation it was discovered to originate from insertion of a mobile genetic element in the locus encoding Wingless (Wg)^29^—the founding member of the Wnt morphogens in *Drosophila* and humans^30^. Wg is a key determinant of pattern formation in *Drosophila* tissues including the eye^9,10^. Mis-expression of Wg in the eye imaginal disc attained in the *Glazed* mutant or upon direct genetic overexpression induces apoptosis of the differentiated eye cells and fusion of ommatidia^27,29^. The similarity of these phenotypes to those induced by expression of human RPS12 indicated that this ribosomal protein might somehow stimulate Wg production or signaling—the hypothesis highly appealing given the key role of Wnt signaling in TNBC from which the transgene originated.

**Figure 4:**
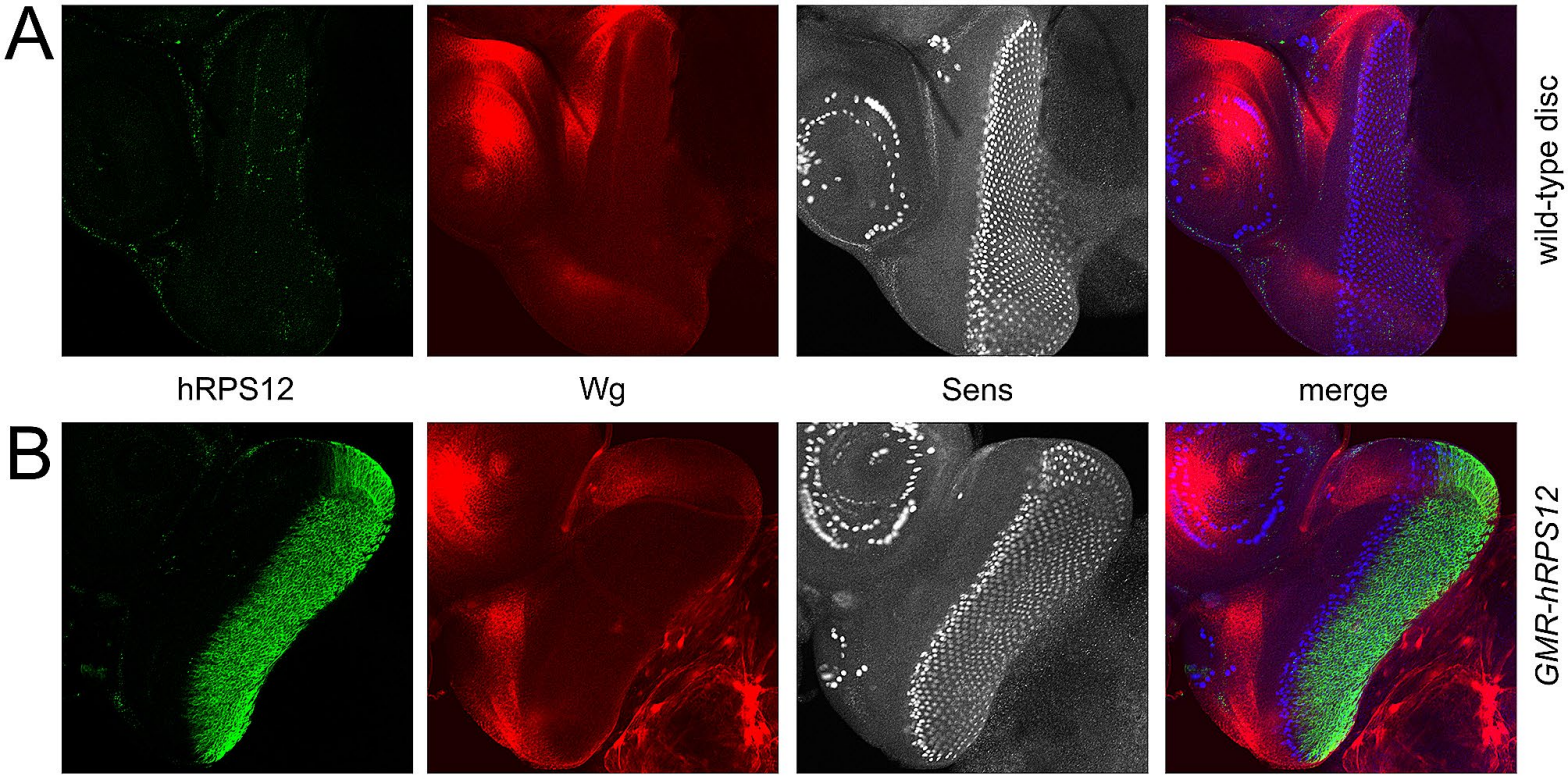
Wg and hRPS12 immunostaining in 3rd-instar larval eye-antennal discs. (**A**) Wild-type disc shows no staining with antibodies against human RPS12. Wg staining is restricted to the periphery of the eye disc and is absent from the domain of cell differentiation marked by expression of Sens. (**B**) *GMR-hRPS12* disc shows prominent expression of hRPS12 colocalizing with the Sens-expression domain; no ectopic Wg expression can be seen.

To test if there is a genetic interaction between hRPS12 and Wg, we expressed an RNAi against Wg on top of *GMR-hRPS12*, producing a remarkable rescue of the eye phenotypes (Fig. 3B,C). In the eye imaginal disc, Wg synthesis is restricted to the periphery of the disc forming the future head capsule^9^ and is largely excluded from the differentiated cells expressing the *GMR-Gal4* driver (marked by expression of the Senseless [Sens] protein in Fig. 4A). Importantly, hRPS12 does not elicit immunostaining-detectable mis-expression of Wg in the differentiated eye cells in the larval stage (Fig. 4B). Low levels of Wg expression have been ascribed to differentiated cells such as cone cells at later stages of the eye development^31,32^, and hRPS12 may amplify the signal emanating from this later Wg production by the differentiated eye cells. Interestingly, we find that inhibition of the Wg signaling pathway by expression of AxinΔRGS, highly potent in other contexts^21^, only modestly rescues the hRPS12 eye phenotypes, unlike the *RNAi-Wg* construct (Fig. 3B,C). These findings suggest that hRPS12 mainly acts at the level of Wg production / secretion / diffusion through the tissue, rather than at the level of Wg signaling in the receiving cells. Similar activity was previously ascribed by us to reggie-1/flotillin-2—a structural protein of membrane microdomains, mediating special packaging of the Wg morphogens for their long-range diffusion through the receiving tissue^14^. In order to investigate whether hRPS12 could indeed influence Wg diffusion, we moved to the tissue more pertinent to analyze this phenomenon, i.e. the wing imaginal disc of *Drosophila*.

### RPS12 expands the Wg diffusion gradient

**Figure 5:**
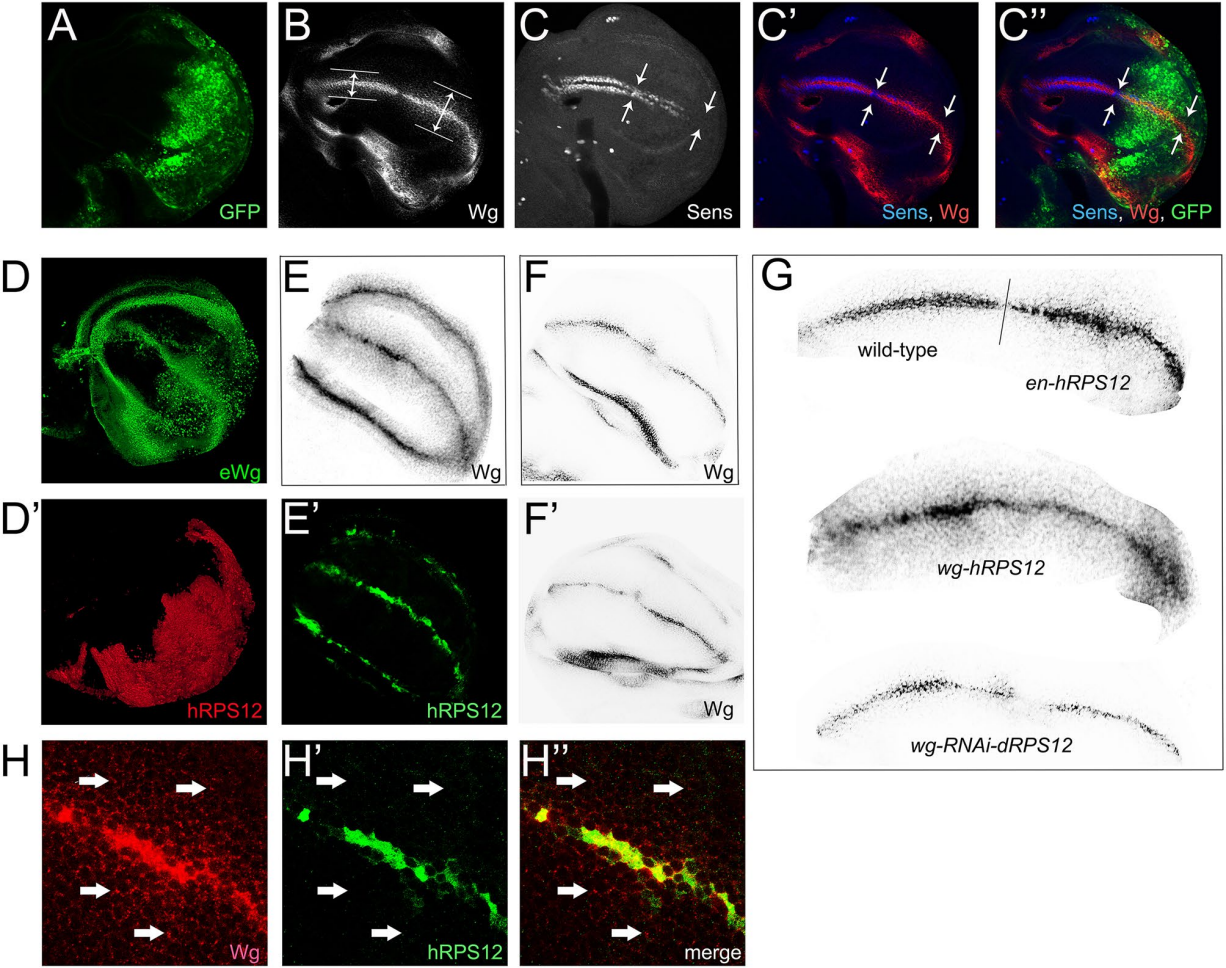
RPS12 affects Wg diffusion in wing imaginal discs. (**A**–**C**) In *en-hRPS12* wing discs, the anterior half of the disc serves as an internal wild-type control to the posterior domain expressing hRPS12 (marked by the co-expressed GFP, **A**, **C**″). The posterior domain displays remarkably enlarged Wg diffusion (**B**,**C**′,**C**″) concomitant with a decreased expression of the short-range Wg target gene Sens (**C**,**C**′,**C**″). (**D**,**D**′) shows another *en-hRPS12* wing disc, where the extracellular Wg (eWg) gradient is visualized. The image is the flattening of multiple recorded Z-stacks. (**E**,**E**′) In *wg-hRPS12* discs, hRPS12 expression is restricted to the stripe of Wg expression at the D/V border of the disc proper, as well as the zone of Wg expression around the wing proper. Such expression of hRPS12 in the Wg-producing cells dramatically expands the resulting Wg diffusion gradient. (**F**,**F**′) The opposite effect on Wg diffusion is observed in *wg-RNAi-dRPS12* discs (two discs are shown, with contrast of the anti-Wg staining in (**F**) identical to that in (**D**,**E**), and with different settings to maximize the Wg signal in (**F**′). (**G**) shows higher magnification of the Wg gradients emanating from the dorso-ventral boundary of discs of the indicated genotypes, taken at identical confocal and contrast settings. (**H**,**H**″) Higher magnification of a *wg-hRPS12* wing disc stained for Wg (**H**,**H**″) and hRPS12 (**H**′,**H**″) shows that the Wg puncta diffusing away from the source of production are devoid of hRPS12.

In wing imaginal discs, overexpression of reggie-1/flotillin-2 e.g. in the posterior compartment of the disc induced expansion of the Wg diffusion in this compartment as compared to the wild-type anterior compartment serving as an internal control; decreased expression of the short-range Wg target gene Sens was also seen in the posterior compartment, resulting from the Wg gradient erosion^14^. We thus aimed at testing whether expression of the human RPS12 in wing discs would produce similar phenotypes. Remarkably, expression of hRPS12 under the control of the posterior *engrailed-Gal4* driver (*en-hRPS12*) marked by the co-expressed GFP (Fig. 5A) indeed eroded the Wg diffusion gradient (Fig. 5B), resulting in decreased expression of Sens (Fig. 5C,C′,C″), which requires high levels of Wg for its proper induction^14^. The Wg gradient erosion was also seen upon hRPS12 expression using another posterior compartment-specific driver line, *hedhehog-Gal4* (data not shown). Importantly, the expansion of the Wg gradient by hRPS12 was also seen upon investigation of the extracellular Wg (Fig. 5D), similarly to the effect of reggie-1/flotillin-2 described previously^14^.

In order to differentiate between the ability of hRPS12 to enhance Wg diffusion from within the Wg-secreting cells—i.e. to affect the form in which the Wg morphogen is produced—and the possibility that hRPS12 acts in the tissue through which Wg diffuses, we expressed hRPS12 exclusively within the Wg-producing cells, using the *Wg-Gal4* driver line (*wg-hRPS12*). We reciprocally down-regulated the endogenous levels of dRPS12 within the Wg-producing cells through expression of an RNAi-construct against *Drosophila* RPS12 (*wg-RNAi-dRPS12*). In this manner, we find that increased RPS12 levels (through expression of the human RPS12) exclusively in the Wg-producing cells markedly enhances (Fig. 5E), while downregulation of dRPS12 decreases, the Wg diffusion properties (Fig. 5F,F′). Side-wise comparison of the Wg diffusion gradients in the control (anterior compartment of the *en-hRPS12* discs), hRPS12-expressing (posterior compartment of the *en-hRPS12* discs and even more evidently—*wg-hRPS12* discs), and dRPS12-downregulated conditions (Fig. 5G) shows a dramatic dependence of the Wg spreading on the RPS12 levels.

These observations are again very reminiscent of those we made upon genetic manipulations of reggie-1/flotillin-2 and suggest that RPS12 possesses a previously undiscovered role in regulating the form in which Wg is secreted, such that more RPS12 makes Wg more diffusive, and less RPS12 makes Wg less diffusive. A further similarity between the reggie-1/flotillin-2 and RPS12 cases is provided upon high-magnification examination of *wg-hRPS12 discs* (Fig. 5H). Specifically, as we previously demonstrated for reggie-1/flotillin-2^13^, hRPS12 expressed in the Wg-production stripe of cells is absent from the Wg foci spreading away from the source of production (arrows in Fig. 5H). Thus, RPS12 stimulates Wg diffusion through the receiving tissue, but itself does not travel together with the morphogen.

## Discussion

RPS12 is a 14 kDa protein belonging to the small ribosomal subunit^33^. It is located on the outer ribosome surface^34^, and its ribosomal presence and content can vary depending e.g. on the cell hypoxia^35^.

Increased expression of hRPS12 has been described in a number of cancers, such as colorectal^36^, gastric^37^ and cervical^38^. In the latter case, hRPS12 was even proposed as a marker for the early cancer detection^39^. Particularly important for our current study, RPS12 was found strongly over-represented in infiltrating ductal carcinoma tumors as compared to the neighboring healthy tissues in multiple breast cancer patients tested, as analyzed by mass-spectrometry; it was the only ribosomal protein identified in this study^40^. Further relevant for the oncogenic growth, RPS12 has been identified as an important player in the phenomenon known as cell competition^41,42^.

Despite these findings, the mechanistic understanding of the role of RPS12 in cancer initiation and progression, which might be decoupled from the ribosomal activity of this protein, is lacking. In our work, we find that RPS12 levels correlate with the secretion / diffusion of the Wnt oncogenes in the *Drosophila* model. Although Wnt signaling is key for excessive proliferation of human breast cancer cells^11,12^, in *Drosophila* eye it restricts the tissue growth^9^, explaining the observation that expression of hRPS12 led to reduced eyes in the fruit fly. Further investigation is required to understand how exactly RPS12 contributes to Wnt production. It might regulate the site (apical or basolateral) of Wnt mRNA translation, which is known to be important for the mode of secretion and subsequent diffusion properties of the morphogen^43–45^. Alternatively, RPS12 may directly contribute to the packaging of Wnts into different vehicles for the long-range diffusion through the tissue, in a manner reminiscent of the activity of the reggie-1/flotillin-2 protein^14^. Finally, RPS12-containing *vs.* RPS12-devoid ribosomes might have differential ability to translate select mRNAs as has been shown in the case of some other ribosomal proteins^46^; among such mRNAs one might anticipate those encoding regulators of Wnt secretion. While the exact mechanism is still unknown, the capacity of RPS12 to promote long-range diffusion of Wnt might be important for tumor cell proliferation and migration, given the key role of Wnt signaling for carcinogenesis in multiple tissues such as breast, colon, stomach, liver, cervix, ovaries, and more^11^.

Identification of RPS12 as an unexpected candidate regulator of Wnt secretion in cancer cells is just the first outcome of the large-scale project we initiated to uncover novel cancer-relevant proteins using the *Drosophila* model. This project we dubbed HumanaFly builds upon the high conservation of pathology-related genes between humans and the fruit fly, on the one hand, and upon the inherent kinship of the embryonic and oncogenic signaling pathways, on the other. Differently from other examples of massive analysis of human genes in *Drosophila* (see e.g.^47^), HumanaFly focuses on identification of the relevant transgenes based on their function. In the run of the project, we aggressively filter out the majority of the cancer transgenes, with only ca. 0.3% retained for subsequent analysis based on their ability to disturb the sensitive multi-component process of the insect’s eye development. The screen we have performed up to now is far from saturation. We expect that continuation of the HumanaFly project will bring several novel and unexpected players in the cancer initiation and progression to the light of scientific investigation and, subsequently, targeted drug discovery.

## Supplementary information

Supplementary Figure 1.
