# Supplementary material for: HumanaFly: high-throughput transgenesis and expression of breast cancer transcripts in Drosophila eye discovers the RPS12-Wingless signaling axis
Source: Sci Rep. 2020 Dec 3;10:21013. doi: 10.1038/s41598-020-77942-x (PMC7713366; doi:10.1038/s41598-020-77942-x)
Supplement: Supplementary file 1 — Supplementary Figure 1. [file 41598_2020_77942_MOESM1_ESM.pdf]

HumanaFly: high-throughput transgenesis and expression of breast cancer transcripts in *Drosophila* eye discovers the RPS12-Wingless signaling axis

Vladimir L. Katanaev[1,2,3]*, Mikhail Kryuchkov[1,2], Volodymyr Averkov[2], Mikhail Savitsky[1], Kseniya Nikolaeva[2], Nadezhda Klimova[2], Sergei Khaustov[2], Gonzalo Solis[1]

[1]Translational Research Center in Oncohaematology, Department of Cell Physiology and Metabolism, Faculty of Medicine, University of Geneva, Geneva, Switzerland;

[2]Developmental Genetics Group, Institute of Protein Research, Russian Academy of Sciences, Pushchino, Moscow Region, Russia;

[3]School of Biomedicine, Far Eastern Federal University, Vladivostok, Russia.

*author for correspondence: vladimir.katanaev@unige.ch

SUPPLEMENTARY INFORMATION FILE, containing Supplementary Figure 1.

Supplementary Figure 1. Drosophila eye phenotypes in the HumanaFly project. (A) Flies used for the germ-line transformation with human breast cancer transgenes have white-colored eyes (genotype: *yw, ZH-attP-22A (φχ22A)*). (B) Upon successful transformation with the pUASTattB vector carrying the *white*+ transgene as a marker, the red eye color is produced. (C, D) The post-injection P0 flies were crossed with the line carrying *GMR-Gal4*, and the transformant lines carrying human breast cancer transgenes, whose expression in the *Drosophila* eye led to morphological aberrations, were maintained for further investigation. The rough-eye phenotype induced by expression of human ANKRD17 (C) and OXA1L (D) is shown.

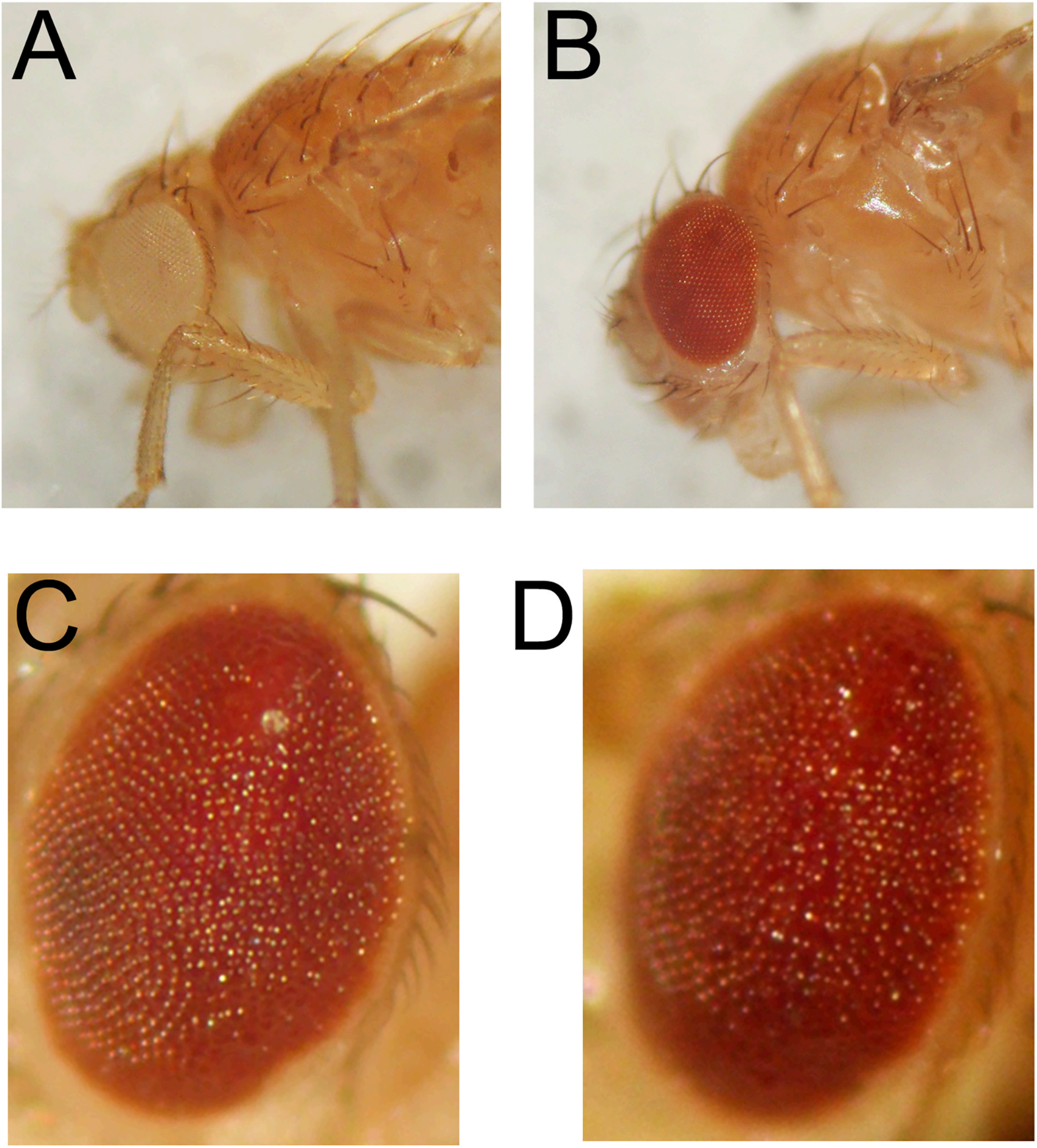
A
B
C
D
